# Ingluvin in the Vomiting of Pregnancy

**Published:** 1889-04

**Authors:** 


INGLUVIN IN THE VOMITING OF PREGNANCY.—DR. POPP (*Pester Med. Presse*, No. 40, 1888,) reports having achieved considerable success with ingluvin in the vomiting of pregnancy. Having a very obstinate case, upon which he had exhausted the entire resources of the pharmacopeia, he administered three times daily, one-half hour before meal-time, eight grains of ingluvin, and directly afterward two tablespoonfuls of one per cent. hydrochloric acid solution. An improvement was observed after a few doses had been taken, and a cure effected after the treatment had been continued for three weeks. —*Deutsche Med. Wochenschrift*, Jan. 17, 1889.

